# *In vitro* Mycobacterial Growth Inhibition in South Korean Adults With Latent TB Infection

**DOI:** 10.3389/fimmu.2019.00896

**Published:** 2019-04-26

**Authors:** Hyejon Lee, Jungho Kim, Young Ae Kang, Deok Ryun Kim, Bora Sim, Andrea Zelmer, Helen A. Fletcher, Hazel M. Dockrell, Steven G. Smith, Sang-Nae Cho

**Affiliations:** ^1^Clinical Vaccine Research Section, International Tuberculosis Research Center, Seoul, South Korea; ^2^Department of Microbiology, Institute of Immunology and Immunological Disease, Yonsei University College of Medicine, Seoul, South Korea; ^3^Division of Pulmonary, Department of Internal Medicine, Severance Hospital, Institute of Chest Diseases, Yonsei University College of Medicine, Seoul, South Korea; ^4^Development and Delivery Unit, International Vaccine Institute, Seoul, South Korea; ^5^Department of Immunology and Infection, Faculty of Infectious and Tropical Diseases, London School of Hygiene & Tropical Medicine, London, United Kingdom

**Keywords:** mycobacterial growth inhibition, correlates, vaccine, tuberculosis, latent tuberculosis infection

## Abstract

**Background:** It is important to understand the ability to inhibit mycobacterial growth in healthy adults who would have been Bacillus Calmette-Guérin (BCG) vaccinated in childhood as this group will be the potential target population for novel booster TB vaccine trials. In this study we investigated not only the long-term immunity induced by childhood BCG vaccination but also protective immunity in terms of the ability to inhibit mycobacterial growth in those who were BCG vaccinated in childhood, with evidence of recent or remote TB infection.

**Methods:** We measured the baseline immune response using a functional mycobacterial growth inhibition assay (MGIA) as a novel approach and an intracellular cytokine staining (ICS) assay as a reference approach in healthy adults, with different status of *Mycobacterium tuberculosis* (Mtb) infection.

**Results:** Based on MGIA responses in historically BCG-vaccinated healthy adults, demographical characteristics including age, and gender did not affect mycobacterial growth inhibition in PBMC. However, the uninfected healthy control (HC) group showed a greater ability to inhibit mycobacterial growth compared with the latent TB infection (LTBI) group (*P* = 0.0005). In terms of the *M. tuberculosis* antigen-specific T-cell immune response in diluted whole blood quantitated using an ICS assay, the LTBI group had a higher frequency of polyfunctional CD 4+ T cells compared with the HC group (*P* = 0.0002), although there was no correlation between ICS and the MGIA assay.

**Conclusion:** The Mtb infection status had a significant impact on mycobacterial growth inhibition in PBMC from healthy adults in South Korea, a country with an intermediate burden of tuberculosis, with healthy controls showing the greatest mycobacterial growth inhibition.

## Introduction

Pulmonary tuberculosis (TB) is an airborne infectious disease caused by *Mycobacterium tuberculosis* (Mtb), which is a significant burden for the world, reaching 9.6 million new TB cases and 1.5 million deaths per year (1). Also, just under a quarter of the global population is latently infected with Mtb, and 5–10% of them will develop TB in their lifetimes (2). Although there has been a noteworthy improvement in controlling TB since the mid-1960s associated with economic growth (3, 4), TB still remains a major public health problem in South Korea (S. Korea), a country with an intermediate burden of TB with an estimated TB prevalence rate of 70.4 per 100,000 population (5). The age distribution of TB in S. Korea shows incidence peaks in those aged between 20 and 30 years of age and the elderly (above 65 years of age) (3, 6, 7). This may indicate ongoing transmission at the community level, as TB cases mainly occurred in the young adult group (following recent infection) and in the old age group (resulting from remote infection and reactivation). Considering the characteristics of TB epidemiology of S. Korea as a mixture, with high prevalence of TB in the elderly and outbreaks in certain populations such as close contacts of patients with TB, health care workers at workplaces, and immigrants from high TB burden countries etc., there is a need for new TB vaccines to control tuberculosis in S. Korea. In line with the goal of the national TB control plan to reach 50% reduction in TB incidence by the year 2020, the introduction of novel TB vaccines to adults together with continued Bacillus Calmette-Guérin (BCG) vaccination to infants might be one of the most effective strategies for S. Korea as well as for other Asian countries in similar situations. However, BCG is the only vaccine available and has been used for more than 90 years. Based on numerous studies, BCG vaccine efficacy varies widely (from 0 to 80%) between geographical locations, with demographical characteristics, and with the socio-economic status of the populations (8, 9). With this partial protective efficacy of BCG in humans, it is important to introduce novel TB vaccine candidates that will work for all ages, regions, and regardless of the HIV epidemic, along with improvement of rapid diagnosis and effective drug treatments. With continuous efforts to overcome the hurdles for effective TB vaccine development including the absence of a predictive animal model or validated correlates of protection, we need to prioritize vaccine candidates that would be effective across the entire spectrum of TB infection ranging from latent TB infection (LTBI) to active TB disease.

In order not to embark on a costly vaccine efficacy trial without a high chance of success, it would be very helpful to have one or more biomarkers as a “correlation of protection.” Coordinated by the TuBerculosis Vaccine Initiative (TBVI), research groups as part of Work Package (WP) #5 in the EU Horizon 2020 TBVAC2020 consortium have been working to develop novel biomarker assays with a common goal to identify, test, evaluate, and prioritize surrogate-endpoints of protection against human TB, which would give us information about vaccine induced immunogenicity and the potential efficacy of novel vaccine candidates at an early stage of the developmental pipeline (10). Recently, *in vitro* mycobacterial growth inhibition assays (MGIA) as functional measures of human protective responses using the commercial BACTEC machine has been standardized, with recommendations made for its use in vaccine and other studies (11–15). A number of different MGIA assays have been used in animal and human studies (13, 15–21). For example, the MGIA can be performed with different cell types (whole blood-based or PBMC-based), using different mycobacteria (BCG or *M. tuberculosis* H37Rv), different protocols (in rotating tubes or in static 48-well plates) and using different study populations with different infection status and geography. The precise mechanisms of mycobacterial growth inhibition remain to be identified, despite a number of studies (10, 12, 22). Compared to classical read-outs such as the IFN-γ Enzyme-Linked ImmunoSpot (ELISpot), or the IFN-γ enzyme-linked immunosorbent assay (ELISA), and intercellular cytokine staining (ICS) flow cytometry assays, a MGIA is a more direct way of assessing anti-mycobacterial immunity. Particularly, the group at the University of Oxford has contributed significantly to the development of the optimized MGIA assay through the TBVAC2020 project, and active collaboration with the European Research Infrastructures for Poverty Related Diseases (EURIPRED) project on standardizing MGIA, ICS and Luminex assays for identification and evaluation of potential immune correlates in humans, non-human primates (NHP) and mice (22–25). The current MGIA protocol uses reference mycobacteria, exploits the commercial BACTEC MGIT 960 system (http://www.euripred.eu/information-trainings/sops-assay-harmonization.html) and was developed from an earlier protocol used for TB drug evaluation (26). Alternative approaches to MGIA have used recombinant BCG such as BCGlux or combinations of antigen-stimulated lymphocytes and monocytes (13, 27, 28), but these assays are more complex and many therefore be less suited for use in vaccine trials. In a joint effort between the research groups of Workpackage 5 in TBVAC2020, and EU-funded consortium aiming to facilitate the development of new TB vaccines, a number of biomarker studies using human MGIA assays have been on-going. For example, Joosten et al. and O'Shea et al. observed strong mycobacterial growth inhibition, compared with other immune assays including ICS and Luminex on the samples from the same clinical cohorts (17, 24). When the MGIA assay was used in studies of BCG vaccinated infants, greater growth inhibition was observed following BCG vaccination than in unvaccinated controls (29). Tanner et al. also developed an *in vitro* model to assess the impact of monocyte phenotype on the ability to control mycobacterial growth, in order to identify immune mechanisms (13). Based on these research activities of a number of research groups using different human cohorts and sample repositories from different settings, standardized MGIA assays can now be used for assessment of mycobacterial growth inhibition in clinical cohorts (23).

Prior to a clinical trial of novel TB vaccine candidates in South Korea, such as the subunit ID-93 vaccine/GLA-SE vaccine candidate, being developed for prevention of TB infection (30–32), this study was carried out to measure baseline immune responses using MGIA assays (as a novel assay) compared with ICS assays (as a reference assay) in healthy adults aged more than 20 years old.

## Materials and Methods

### Study Participants and Sample Collection

Healthy adults aged more than 20 years old were recruited from the Severance Hospital, Seoul, S. Korea between January 2016 and September 2017. Individuals with any acute or chronic disease, with a previous history of active TB, or with any suggestive symptoms of TB were excluded. BCG vaccination status was also assessed based on the survey as well as on-the-spot inspection of BCG scar on the left or right upper arm. However, >90% of adults in S. Korea would have been BCG vaccinated in childhood as per the vaccination policy regardless of the presence of a BCG scar(s). The status of Mtb infection was assessed using the QuantiFERON-TB® Gold In-tube assay (QFT). None of the participants in this study had HIV infection; a chronic comorbidity such as diabetes mellitus, chronic renal failure, malignant tumors, or chronic liver disease; immunosuppressive status; or acute infections. This study was approved by the Institutional Review Board of Severance Hospital (IRB No. 4-2014-1108). All participants provided written informed consent to use their clinical information and specimens for evaluation of TB biomarkers. Peripheral blood from all participants was collected into sodium heparin tubes (Becton, Dickinson (BD), New Jersey, USA) for QFT, MGIA, and ICS assays.

The *Mycobacterium bovis* BCG (Pasteur 1173P2) batch used was prepared and provided by the Aeras Foundation after testing for “viability and reproducibility” prior to distribution through the TBVAC2020 partnership (12).

### QFT

At baseline all participants were tested using a TB-specific interferon gamma release assay, the QFT assay (Qiagen, Hilden, Germany) performed according to the manufacturer's instructions. Briefly, 1 mL of whole blood was collected in each of three tubes pre-coated with *Mycobacterium tuberculosis*-specific peptides (ESAT-6, CFP-10, and TB7.7) or mitogen (positive control) and incubated for 16–24 h at 37°C. For negative controls, whole blood was placed in tubes that were not pre-coated. The plasma supernatant was harvested by centrifugation at 3,000 × g for 15 min and stored at −80°C. The concentration of IFN-γ was determined using the QFT Enzyme-linked Immunosorbent Assay (ELISA) Kit. All quantitative QFT test results were expressed as IFN-γ concentration (IU/mL) and qualitative QFT test results were interpreted according to the manufacturer's algorithm using QFT software version 2.62.

### PBMC Cryopreservation and *in vitro* Mycobacterial Growth Inhibition Assay

Heparinized blood (> 10 mL) collected in a cell preparation tube (CPT; Novamed, Jerusalem, Israel) was centrifuged at 1,150 × g for 20 min without deceleration. The peripheral blood mononuclear cell (PBMC) layer was transferred to a fresh tube and washed 2 times with Hanks' balanced salt solution (HBSS; Welgene, Gyeongasn, Republic of Korea). After adding one milliliter of 1 × Pharm-Lyse solution (BD Biosciences, New Jersey, USA), the PBMCs were incubated for 5 min at room temperature to disrupt erythrocytes. After washing, the PBMCs were frozen in fetal bovine serum (FBS; Invitrogen, Massachusetts, USA) with 10% dimethylsulfoxide (DMSO; Sigma, Missouri, USA) and stored at −80°C for 24 h before transferring to liquid nitrogen until used. Cryopreserved PBMCs were thawed and rested for 2 h at 37°C in RPMI 1640 (Hyclone, Logan, UT, USA) with 10% FBS and 10 units/mL of benzonase (Novagen), then washed and re-suspended in RPMI 1640 with 25 mM HEPES supplemented with 2 mM L-glutamine and 10% filtered, heat-inactivated, pooled human AB serum (Sigma). One million of PBMC (1 × 10^6^) were co-cultured in 2-mL screw-cap tubes for 4 days with 100 colony forming units (CFU) of BCG Pasteur at 37°C with 360° rotation in a final volume of 600 μL. All samples were run in duplicate. Following incubation, 600 μL samples were transferred into a Mycobacterium Growth Indicator tube (MGIT; BD, New Jersey, USA) supplemented with PANTA enrichment, and the tube was placed in a BACTEC MGIT 960 machine (BD, New Jersey, USA) and incubated until mycobacterial growth rate was determined by the time to positivity (TTP) as previously described (13, 22). As a control, called “Direct-to-MGIT,” in which the bacterial inoculum being used in the tubes is added directly to a MGIT tube, duplicate MGIT tubes seeded with the sample concentration (100 CFU) of BCG Pasteur without added cells were placed directly in the BACTEC MGIT 960 machine on day 0.

### Diluted Whole Blood Intracellular Cytokine Staining (ICS) Assays

As described by Smith et al. (29), venous blood (1 mL) was diluted 1:1 with warm Iscove's modified Dulbecco's medium (IMDM; Invitrogen, Massachusetts, USA) in 15 mL centrifuge tubes (HYUDAI Micro, Seoul, Republic of Korea). Diluted blood was incubated with medium only as a negative control or with 10 μg/mL of Mtb purified protein derivate (PPD) for *in vitro* use (Statens Serum Institute, Copenhagen, Denmark) or with 5 μg/mL staphylococcus enterotoxin B (SEB; Sigma) as a positive control. Co-stimulatory antibodies (2 μg/mL each of anti-CD28 and anti-CD49d (BD Biosciences) were added to all tubes (33). Assay tubes were incubated with loose lids for 2 h at 37°C after which 3 μg/mL of brefeldin A (Sigma) was added to all tubes. Following further 18 h incubation at 37°C, 100 μL of 20 mM ethylenediamine-tetra acetic acid (EDTA, Sigma) was added and incubated at room temperature for 15 min. Diluted blood was then incubated with 10 volumes of 1 × FACS Lysing Solution (BD Biosciences) at room temperature for 10 min to lyse red blood cells, centrifuged and washed with 3 mL PBS, and pelleted cells were stored in FBS with 10% DMSO at −80°C for 24 h before transferring to liquid nitrogen until assayed. After thawing and washing with 3 mL PBS, cells were surface stained with anti-CD4-APC-H7 (BD Biosciences), anti-CD19-efluor450, and anti-CD14-efluor450 (eBiosciences, California, USA) for 30 min at 4°C. After washing in PBS/0.1% BSA/0.01% sodium azide, cells were permeabilized and fixed with Cytofix/Cytoperm reagent (BD Biosciences) at 4°C for 20 min washed in Perm Wash buffer (BD Biosciences) and stained with anti-CD3-Horizon-V500, anti-IL-2-FITC, anti-TNFα-PE-Cy7 (BD Biosciences), and anti-IFNγ-PerCP-Cy5.5 (Biolegend, California, USA) for 30 min at room temperature. Cells were then resuspended in 250 μL 1% paraformaldehyde (Sigma) and analyzed within 24 h after staining. Data was acquired using an LSR 4-laser Fortessa flow cytometer (BD Biosciences) and FACSDiva acquisition software (BD Biosciences). Analysis was performed using FlowJo (TreeStar Inc., Oregon, USA).

### Statistical Analysis

GraphPad Prism 6 software (La Jolla, CA, USA) was used to perform statistical analyses as described in the figure legends. Mean and SD were calculated for continuous measures, and the Fisher's exact test was used for dichotomous measures. Statistical comparisons between study groups were made using the Mann-Whitney *U*-test and Fisher's exact test. Associations between growth inhibition and ICS responses were measured using Spearman's rank correlation coefficient (r). All *P*-values were two-sided, and *P* < 0.05 was considered to be statistically significant. To determine the relationship between age and QFT test results of study participants, we employed a Generalized Additive Model (GAM) with logit link function with P-spline smothers (Supplementary Figure 1) (34). For ICS assay, the data was analyzed using FlowJo software version 9.1 (TreeStar Inc., Ashland, OR) and SPICE version 5.1 (freely available from http://exon.niaid.nih.gov/spice/). Samples were gated sequentially on singlet, CD14-CD19-, lymphoid, CD3+CD4+ cells and negative control stimulation tubes were used to set cytokine gates (Supplementary Figure 2).

## Results

### Study Participants

We enrolled 121 Korean healthy adults aged more than 20 years old having a normal chest X-ray and no history of tuberculosis. The mean age of all the study participants was 35.1 (SD ±11.4) years, 26.4% (*n* = 32) were male, and 69.4% had detectable BCG scars. The mean ages of the QFT positive (42.3 years ±13.6) and QFT negative groups (32.1 years ± 8.9) were statistically different (*P* = 0.0001). However, the data shows that other general characteristics of the participants including gender and presence of a BCG scar did not affect the result of the QFT-GIT test (Table 1).

**Table 1:** General characteristics of study participants by QFT-GIT assay.

QFT-GIT assay^*^	***n* (%)**	**Age**	**Gender**	**Presence of a BCG scar(s)**
		**Mean years ± S.D**.	**Male, *n* (%)**	**Yes, *n* (%)**
Positive (IFN-γ ≥ 0.35 IU/ml)	35 (28.9)	42.3 ± 13.6	12 (34.2)	21 (60.0)
Negative (IFN-γ < 0.35 IU/ml)	86 (71.1)	32.1 ± 8.9	20 (23.2)	63 (73.2)
*P*-value		0.0001^a^	0.2571^b^	0.1921^b^
All	121 (100)	35.1 ± 11.4	32 (26.4)	84 (69.4)

* *QFT-GIT assay: The QuantiFERON-TB Gold In-Tube, with the results defined as per manufacturer's guideline*.

a Mann-Whitney test;

b *Fisher's exact test*.

### *In vitro* Mycobacterial Growth Inhibition Ability in Healthy South Korean Adults

As shown in Table 1, there was a statistical difference in mean ages between the QFT positive and negative groups using this IGRA assay which aids in diagnosing Mtb infection (*P* = 0.0001). This indicates that age may affect the Mtb infection status as found in Korean National TB data (3, 6).

**Figure 1:**
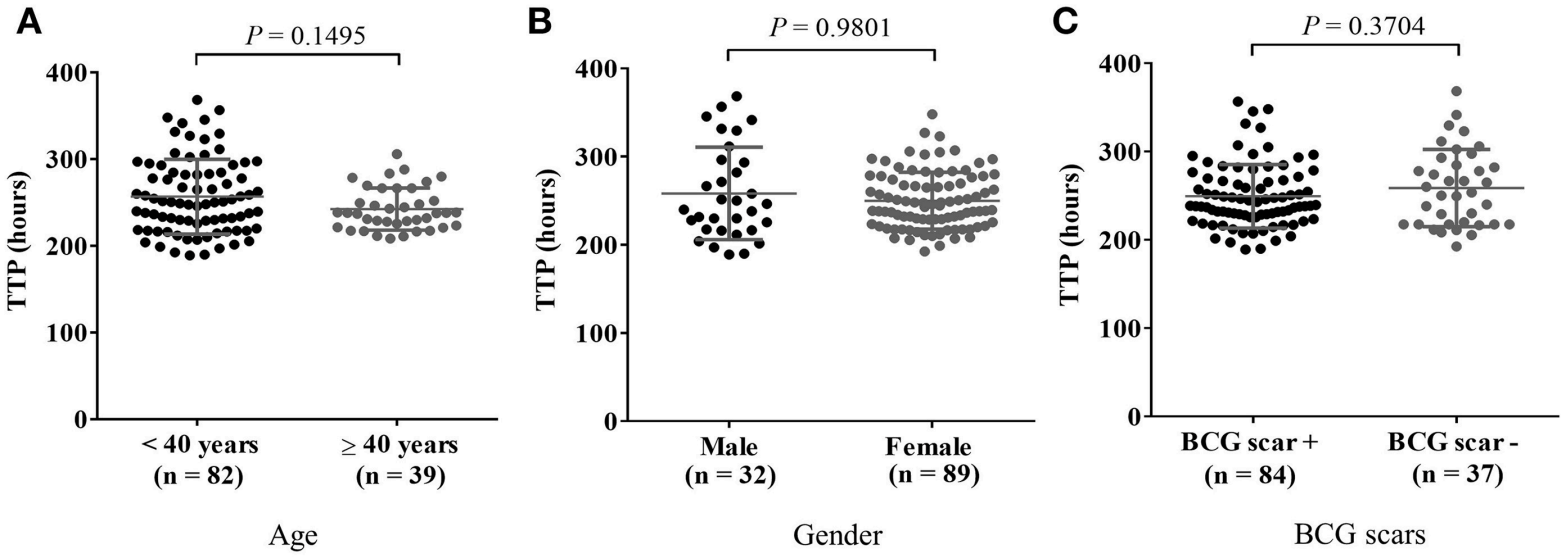
MGIA results in comparison with general characteristics of participants. Cryopreserved PBMC prepared from blood samples from 121 healthy adults were thawed and incubated with BCG for 4 days after which remaining mycobacteria were quantified using BACTEC MGIT tubes. *In vitro* mycobacterial growth in the PBMC based-MGIA assay is presented in panel **(A)** by age group, in **(B)** by gender, and in **(C)** by presence of a BCG scar(s) in healthy adults. A Mann-Whitney *U*-test was used for comparison of the two groups. *P*-value ≤ 0.05 was taken as statistically significant. The growth of BCG Pasteur was plotted as TTP (Time to Positive) in hours.

For subsequent analysis of MGIA results, the data was categorized at cut-off age of 40 years by the risk for Mtb infection based on the relationship between QFT results and age in study participants (Supplementary Figure 1). For the results of the PBMC-based MGIA response analyzed by demographic characteristics of study participants, no significant difference in TTP by age group, gender or BCG status was observed as shown in Figures 1A–C.

For MGIA assays, mycobacterial growth in each tube was determined by TTP in hours. MGIA assays were run on 16 separate occasions to examine the repeatability and intermediate precision, TTP hours from direct-to MGIA tubes as controls were compared (mean 219.7 h ± 15.3). The intra-assay (within-run precision or repeatability) and inter-assay (between-run precision or inter precision) precision coefficients of variation (% CV) were determined to be 2.92 and 6.44%, respectively (Supplementary Figure 3).

### MGIA Response Analyzed by the Status of TB Infection in Healthy Korean Adults

**Figure 2:**
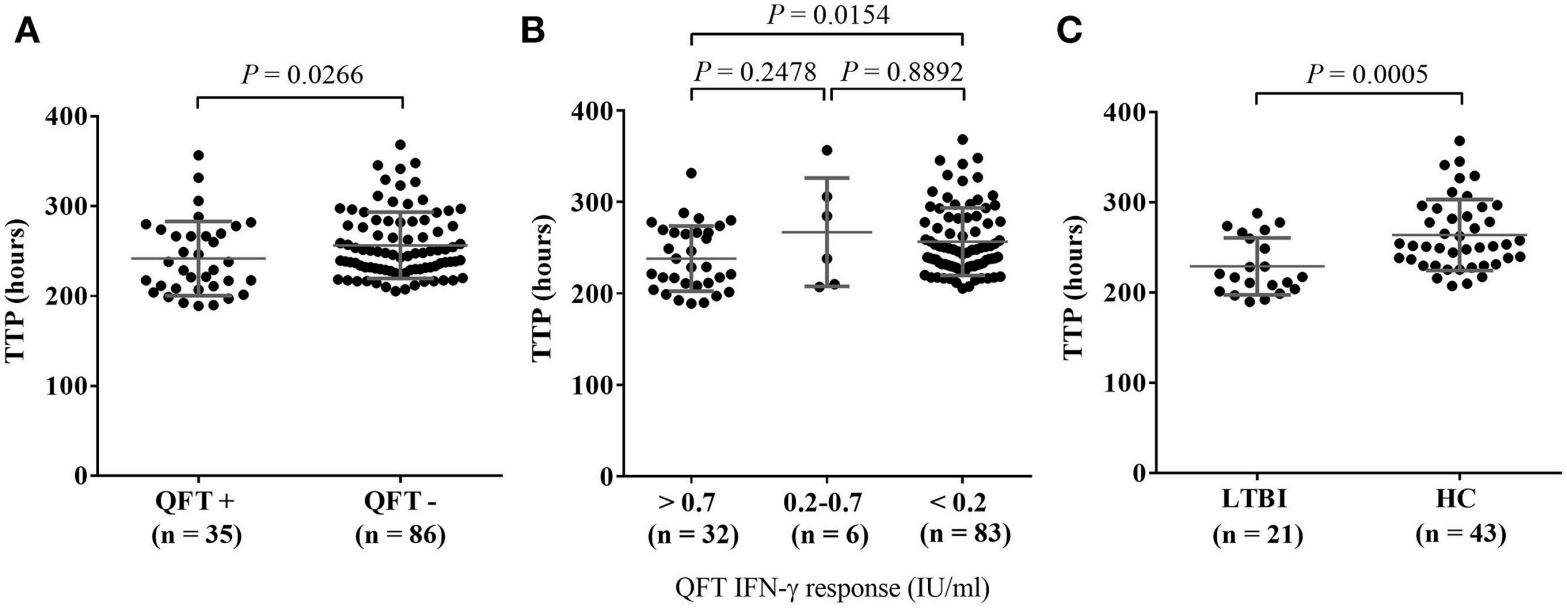
Comparison of MGIA response as TTP (Time to Positive) in hours stratified by QuantiFERON-TB Gold In-Tube (QFT) IFN-γ values in LTBI or HC individuals. MGIA responses of healthy adults (*n* = 121) were stratified by QFT IFN-γ values at baseline. **(A)** Comparison of MGIA response as TTP in hours between the QFT+ (positive test results) and QFT- (negative test results) groups, according to the manufacturer's assay using < 0.35 IU/mL and > 0.35 IU/mL IFN-γ values; **(B)** MGIA response as TTP in hours, stratified by the three categories of IFN-γ values: > 0.7 IU/mL, between 0.2 and 0.7 IU/mL, and < 0.2 IU/mL; **(C)** Comparison of MGIA response as TTP in hours between individuals with latent TB infection (LTBI) defined as QFT + with known contact history with TB patients and healthy controls (HC) defined as QFT—without recent contact history with known TB patients. *P*-values were calculated using Mann-Whitney *U*-test.

MGIA responses expressed in TTP were compared between the QFT positive and negative test groups using a dichotomous cutoff at 0.35 IU/mL as recommended by the manufacturer. The data shows that individuals with QFT negative results had significantly longer TTP in hours showing that those with QFT negative results had a better ability to inhibit the growth of BCG (*P* = 0.0266, Figure 2A). Regarding the reliability of QFT testing for the diagnosis of latent TB infection, several studies have demonstrated that the results of serial QFTs were often not consistent possibly due to immunological and technical variability, and in longitudinal studies both QFT conversions and reversions can be observed (35).

For better interpretation of QFT results, Nemes et al. recently proposed three categories of QFT IFN-γ values: < 0.2 IU/mL as true negatives, 0.2–0.7 IU/mL as an uncertainty zone, and > 0.7 IU/mL as true positives. Based on these three categories of QFT IFN-γ values, MGIA responses (in TTP hours) were compared. Individuals with IFN-γ values < 0.2 IU/mL had significantly longer TTP than those with IFN-γ values > 0.7 IU/mL indicating that the QFT true negatives (< 0.2 IU/mL) had better ability to inhibit the growth of BCG (*P* = 0.0154, Figure 2B).

**Figure 3:**
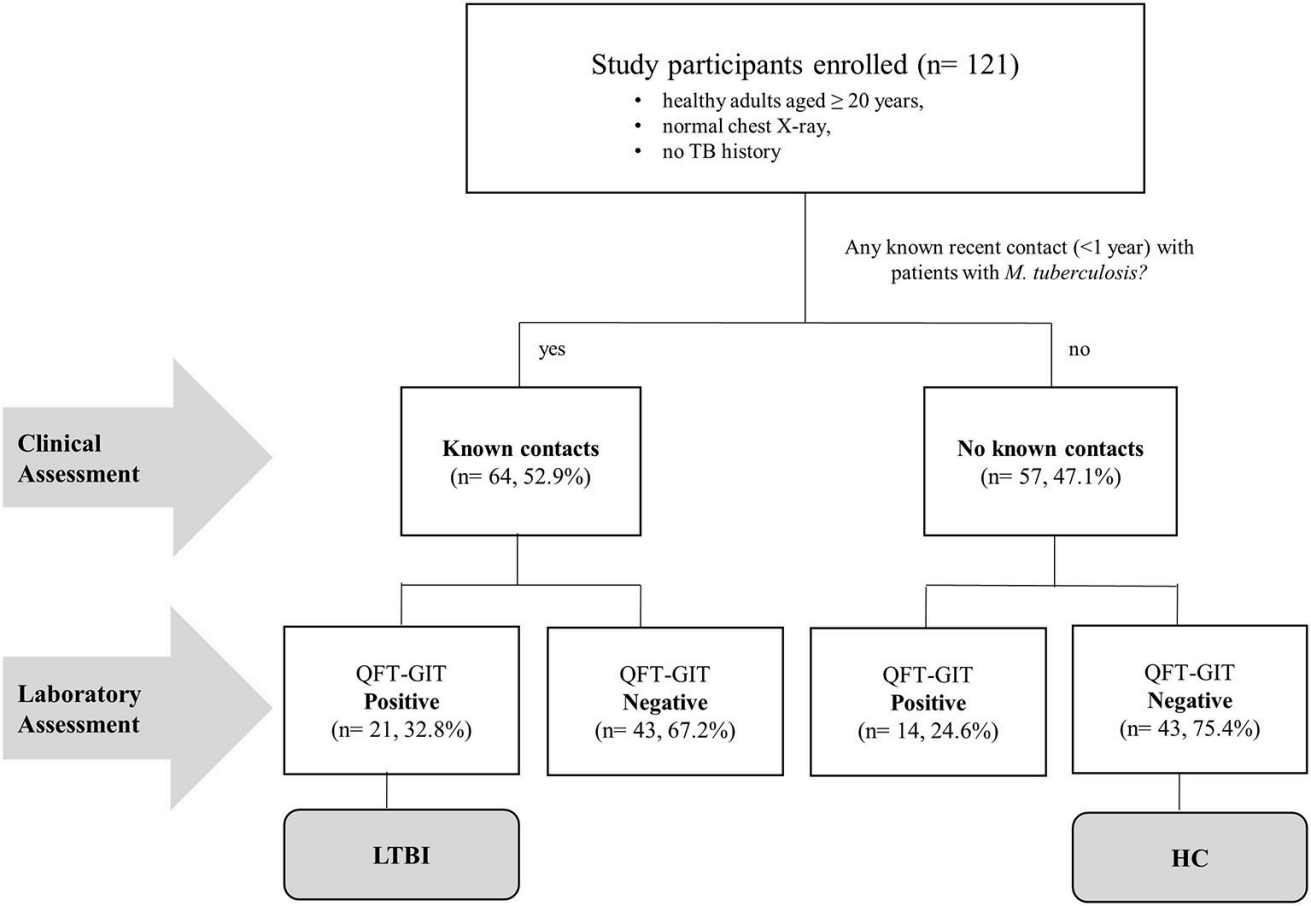
Flow chart of study participants enrolled. A total of 121 Korean healthy adults aged more than 20 years old with normal chest x-ray results and without any tuberculosis history agreed to participate in the study at the Severance Hospital, Seoul. Sixty-four participants had had known contacts with TB patients within the past year. The other participants had had no known previous contacts with TB patients. In addition to clinical evaluation in terms of exposure to *M. tuberculosis*, we tested all study participants with QFT-GIT test for diagnosis of TB infection. Of the known TB contacts, 32.8% (*n* = 21) were QFT positive and defined as individuals with LTBI. In those without known TB contact, 75.4% (*n* = 43) were QFT negative, and were defined as healthy controls (HC) in this study.

In addition, we grouped study participants into LTBI or healthy control (HC) groups, based on clinical as well as laboratory evaluation. Over half (52.9%, *n* = 64) of the study participants had recent contact with a known TB patient within the last year and were defined as a “known TB contact.” The rest of the participants (*n* = 57) who had no recent contact with known TB patients were defined as “no known TB contact.” In addition to clinical evaluation in terms of exposure to Mtb we tested all study participants with the QFT assay. Of the known TB contacts, 32.8% (*n* = 21) were QFT positive and were defined as individuals with LTBI. Of those without any known TB contact, 75.4% (*n* = 43) were QFT negative, and were defined as healthy controls in this study (Figure 3). The MGIA response was compared between the LTBI and HC groups. The data shows that there was significantly greater reduction in growth of mycobacteria in HCs without known TB contact who had longer TTP than in those with LTBI and known TB contact (*P* = 0.0005, Figure 2C).

From observation of these MGIA responses in healthy adults, it appears that the status of TB infection in S. Korea, an area with an intermediate burden of tuberculosis, seems to affect the results of PBMC-based *in vitro* MGIA, demonstrating a greater ability to inhibit the growth of BCG in healthy controls than those with LTBI.

### Relationship Between MGIA and ICS Assays and the Status of TB Infection

**Figure 4:**
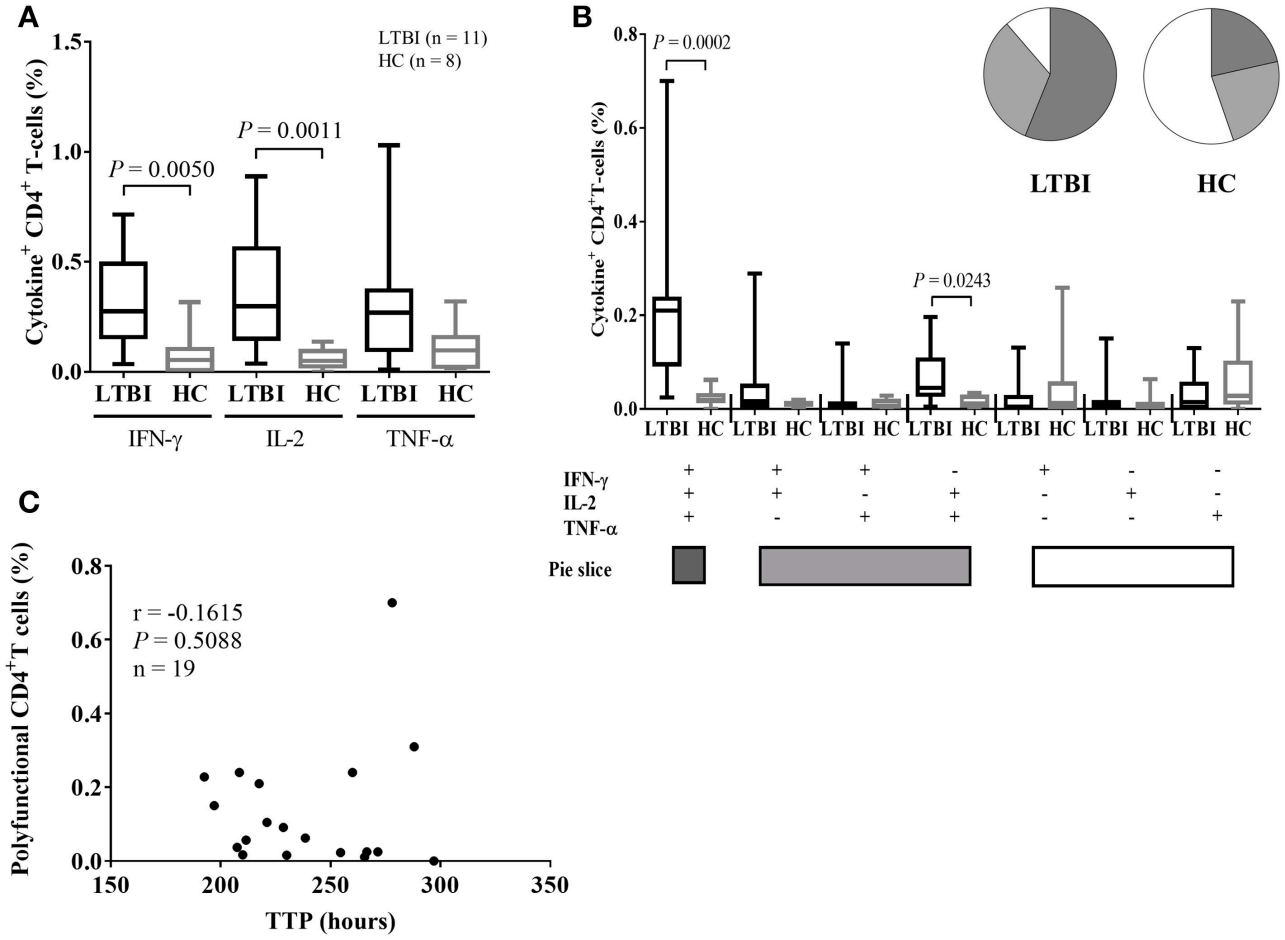
ICS data in LTBI and healthy control groups and correlation between mycobacterial growth inhibition and ICS responses. **(A)** Single cytokine expression data including IFN-γ, IL-2, and/ or TNF-α for individuals with LTBI (black bars, *n* = 11) and healthy controls (gray bars, *n* = 8). The PPD-specific CD4+ T-cell populations making IFN-γ and IL-2 are dominant in the LTBI group. **(B)** The polyfunctionality of CD4+ T-cell populations in individuals with LTBI (black bars) and HCs (gray bars). The PPD-specific polyfunctional T helper-1 cells are the dominant cell type in the LTBI group. Whole blood samples obtained from healthy adults were stimulated with PPD overnight and CD4+ T-cells assessed for cytokine secretion by intracellular staining. All ICS data is plotted as bars showing medians and interquartile ranges. Whiskers show the range of the data. The Mann-Whitney *U*-test was used to determine significance. **(C)** Scatter plots of TTP in hours vs. the percentage of polyfunctional CD4+ T-cells were drawn for all samples where both the mycobacterial growth inhibition assay (MGIA) and the ICS assay were performed (*n* = 19). Inhibition of mycobacterial growth (of BCG Pasteur) is indicated by TTP in hours for the remaining mycobacteria after incubation with PBMC obtained from study participants. The Mann-Whitney *U*-test was used to compare growth inhibition ability between the LTBI and HC groups. Spearman's rank correlation coefficient was calculated between growth inhibition and ICS data, indicated as r.

In this study, based on a diluted whole blood ICS assay, we also measured the frequency of PPD-specific CD4+ T-cells producing IFN-γ, TNF-α, and IL-2 in nineteen study participants. Median cytokine responses in negative control tubes, as a percentage of the gated CD4^+^ T-cell population, were as follows: IFN-γ: 0.044%; TNF-α: 0.075%; IL-2: 0.028%. Median cytokine responses in positive control tubes (SEB-stimulated) were as follows: IFN-γ: 1.810%; TNF-α: 3.790%; IL-2: 4.050% (Supplementary Figure 4). The CD4+ T-cell populations producing IFN-γ and IL-2 were more dominant in the LTBI group than the HC group, respectively (*P* = 0.0050, *P* = 0.0011, Figure 4A). Also, the frequency of PPD-specific IFN-γ+IL-2+ TNF-α+ polyfunctional CD4+T-cells were dominant in the LTBI group (*P* = 0.0002, Figure 4B). These data supported that infection with Mtb activates an antigen-specific, polyfunctional Th-1 response in this study population. However, scatter plots of MGIT TTP in hours vs. the frequency of polyfunctional CD4+ T-cells shows growth inhibition ability did not correlate with PPD-specific polyfunctional CD4+ T cells in the samples (*n* = 19) where both MGIA and ICS assays were performed (Figure 4C). CD4+ T cells producing single cytokines also did not correlate with TTP (Supplementary Figure 5). When the association between the frequency of polyfunctional CD4+ T-cells and MGIT TTP was analyzed separately by the category of QFT values (true negatives, uncertainty zone, and true positives), there also was no correlation between ICS and MGIA assay (results not shown). Higher PPD-specific, polyfunctional Th-1 responses in whole blood did not predict any enhancement of MGI ability in PBMC.

## Discussion

According to a Korean national annual report in 2016, the TB incidence rate increased in young Koreans in the 15–19 age group and showed a peak response in those over 65 years in S. Korea. The proportion of TB cases among immigrants has been increasing from 2.5% in 2011 to 6.9% in 2016 (6). These national statistics indicate that there must be ongoing transmission in group settings such as schools, military settings and workplaces, and also there must be reactivation of TB in the elderly. Besides childhood BCG vaccination, innovative efforts for new adult TB vaccination need to be evaluated to control adult TB in S. Korea. Prior to human trials for novel TB vaccine candidates, it is essential to understand the baseline immune responses of potential vaccine target populations in particular.

Based on the MGIA response in healthy adult controls, demographical characteristics of the healthy adults including age group, gender, and the status of historical BCG scar did not affect the result of mycobacterial growth inhibition in PBMC (Figure 1). However, the QFT negative group (QFT IFN-γ < 0.35 IU/mL) revealed longer TTP, showing that those individuals had a better ability to inhibit mycobacterial growth compared to the QFT positive group (*P* = 0.0266) in this assay using PBMC. This result was reaffirmed by subdividing those tested into three categories of QFT IFN-γ values (< 0.2 IU/mL, between 0.2 and 0.7 IU/mL, and > 0.7 IU/mL); the greater the IFN-γ response to TB specific antigens was, the less was their ability to inhibit mycobacterial growth (*P* = 0.0154). Moreover, the difference in MGIA response was greatest when healthy adults were categorized into LTBI and HC groups more stringently, based on clinical (contact history) and laboratory assessment (QFT testing). The HC group without known TB contact revealed significantly longer time to culture positive hours indicating this group had better ability to inhibit mycobacterial growth compared with the LTBI group who had known recent TB contact (*P* = 0.0005). These results seemed to be contradictory to other recent data (17) where the LTBI group as a whole showed no difference from healthy controls unless exposure was more recent, in which case, growth inhibition was better than the healthy control group. O'Shea et al. also noted that samples from those with active TB disease had better growth inhibition of mycobacteria than LTBI, and that those with LTBI had better growth inhibition of mycobacteria than healthy controls, suggesting that the immune response becomes more effective during active disease or recent infection (24). One reason that might explain this difference in MGIA response in these different studies might be differences between the characteristics of the groups recruited in the different studies. Our study defined “healthy controls” as all Korea-born adults with no known contacts with an active TB patient, negative QFT result, and with a history of BCG vaccination. On the other hand, healthy controls in the study by Joosten et al. were BCG naïve, recruited in the Netherlands and Norway where the burden of TB is low and childhood BCG vaccination is not a part of the national immunization program. Healthy controls living in a country with an intermediate burden of TB and with different environmental exposure to non-tuberculous mycobacteria may have a different ability to control mycobacterial growth in the body than those living in European settings. Another reason to explain this difference in MGIA response might be the different cellular and non-cellular components in whole blood containing neutrophils, antibodies, and complement, which would not be present in the assays using PBMC (36–39). Our study performed *in vitro* experiments with frozen PBMC while the results from the O'Shea group used a whole blood MGIA, and hematological characteristics of the different cell types could affect the results of the assay (25). There may also be differences in the proportions of classical: non-classical monocytes in different populations and settings, which may impact on growth inhibition as CD14 low non-classical monocytes have been shown to be associated with growth inhibition (17).

This study demonstrated that infection with Mtb activates an antigen-specific, polyfunctional Th-1 response as the magnitude of PPD-specific IFN-γ+ IL-2+ TNF-α+ polyfunctional CD4+T-cells was greatest in the LTBI group (*P* = 0.0002) in comparison with the HC group. However, the PPD-specific, polyfunctional Th-1 response in whole blood was not associated with mycobacterial growth inhibition ability in PBMC. These results support previous findings that a positive antigen-specific T-cell immune response does not correlate with the functional immune response measured in the MGIA (17, 40). The only study where the frequency of antigen-specific polyfunctional CD4 T-cells was correlated with *in vitro* mycobacterial growth inhibition was performed in BCG-vaccinated infants in the UK (29), this may reflect differences between adults and the infant cohort that was used in that study.

As a result of collaboration efforts in the TBVAC2020 consortium, we now have standardized and improved protocols available for MGIA as a novel assay and ICS as a reference assay that can be used in TB vaccine trials in S. Korea. As shown in previous studies (14, 15, 29), we found that the current MGIA assay was reproducible and reliable with 2.92% of the intra-assay and 6.44% of the inter-assay in this study (Supplementary Figure 3), within the limits set for such biological assays that would be used in clinical trials. A MGIT assay using frozen PBMC as performed here is more suitable for use in a vaccine trial than a whole blood assay, as frozen PBMC collected before and at different time points post-vaccination can be directly compared in the same assay.

We performed a cross-sectional study to measure baseline immune response in terms of WB-based ICS and PBMC-based MGIA from Korean healthy adults. To our knowledge, the current study demonstrated the first observation of the PBMC-based MGIA in healthy adults exclusively recruited from S. Korea, a country with an intermediate burden of tuberculosis. Given the population/geographical differences in BCG vaccine efficacy (9) and BCG-induced immune responses (41, 42) previously seen, it will be important to evaluate novel and existing biomarker assays in diverse cohorts in order to take these immunological differences into account. This study aimed to assess how the MGIA assay performed in South Korea. The assay has now been used in studies performed in the UK and the Netherlands (17, 24) but more work is needed to compare the performance of the MGIA assay using the now standardized SOP, in other settings and ethnic groups. For further investigations, we also plan to perform the Mtb-based MGIA assay in TB patients, who will be the target population for therapeutic TB vaccination. The use of Mtb rather than BCG might be important to monitor treatment efficacy after drug therapy in parallel with therapeutic vaccination, and we plan to use Mtb in these studies.

In conclusion, Mtb infection status had a significant impact on the mycobacterial growth inhibition ability tested using PBMC from individuals in S. Korea. More work is required to define the key components of the mycobacterial growth inhibition observed. However, the assay proved robust and reproducible and can be used to assess the induction of mycobacterial growth inhibition in clinical trials of new TB vaccines.

## Ethics Statement

All study participants provided written informed consent. The Institutional Ethics Committee of Yonsei University Severance Hospital approved this study (approval #4-2014-1108).

## Author Contributions

HL and S-NC designed the study. HL, JK, SS, HD, and HF were responsible for analysis and interpretation of data. JK and BS conducted experiments. DK and JK provided statistical advice. YK was responsible for the enrolment of participants and reviewed clinical data. HL, JK, SS, HD, HF, AZ, DK, YK, and S-NC were responsible for standardization of assays and drafting of the manuscript. HL, SS, HD, and S-NC were responsible for obtaining funding. All authors read and approved the final manuscript.

### Conflict of Interest Statement

The authors declare that the research was conducted in the absence of any commercial or financial relationships that could be construed as a potential conflict of interest.
