# Supplementary material for: In vitro Mycobacterial Growth Inhibition in South Korean Adults With Latent TB Infection
Source: Front Immunol. 2019 Apr 26;10:896. doi: 10.3389/fimmu.2019.00896 (PMC6497970; doi:10.3389/fimmu.2019.00896)
Supplement: Supplementary file 1 [file Data_Sheet_1.docx]

Supplementary Material

***In vitro* mycobacterial growth inhibition in South Korean adults with latent TB infection**

Hyejon Lee^1, 2†,^ *****, Jungho Kim^1, 2†^, Young Ae Kang^3^, Deok Ryun Kim^4^, Bora Sim^2^, Andrea Zelmer^5^, Helen A. Fletcher^5^, Hazel M. Dockrell^5^, Steven G. Smith^5^ and Sang-Nae Cho^1, 2^

^†^These authors are contributed equally to this work.

*** Correspondence:** Hyejon Lee: [hyejonlee@gmail.com](mailto:hyejonlee@gmail.com)

**1. Supplementary Figures**

**
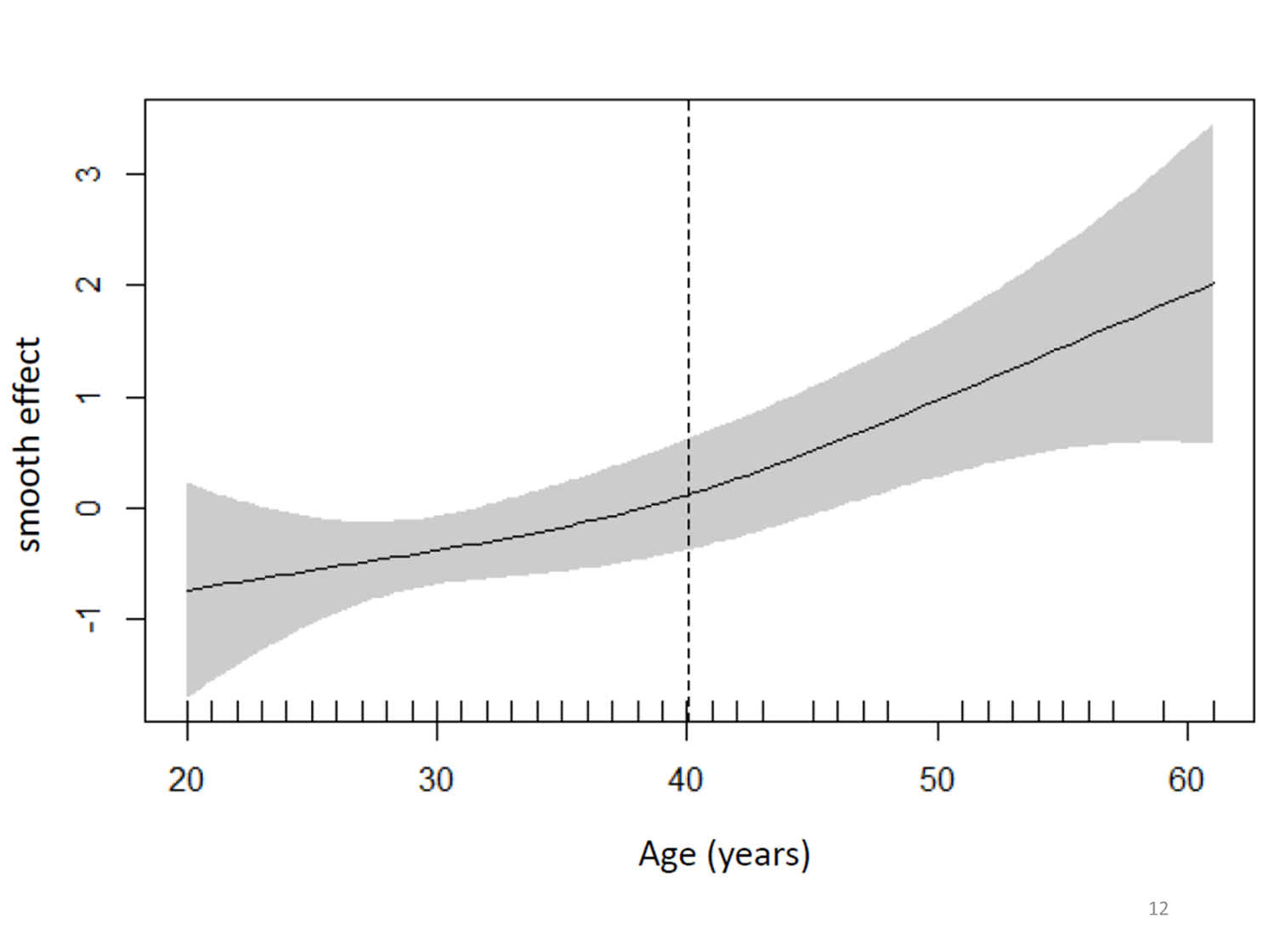
**

**Supplementary Figure 1.** Age threshold of QFT results of BCG-vaccinated Korean adults. The graph shows the estimated smooth relationship of the predictor variable QFT results with the age threshold among Korean healthy adults. A Generalized Additive Model (GAM) with logit link function with P-spline smoothers was employed to determine the relationship between age and QFT results. The analysis suggested that the optimal cut-off is 40.03 years and that the area of age > 40 years was a high risk category.

**
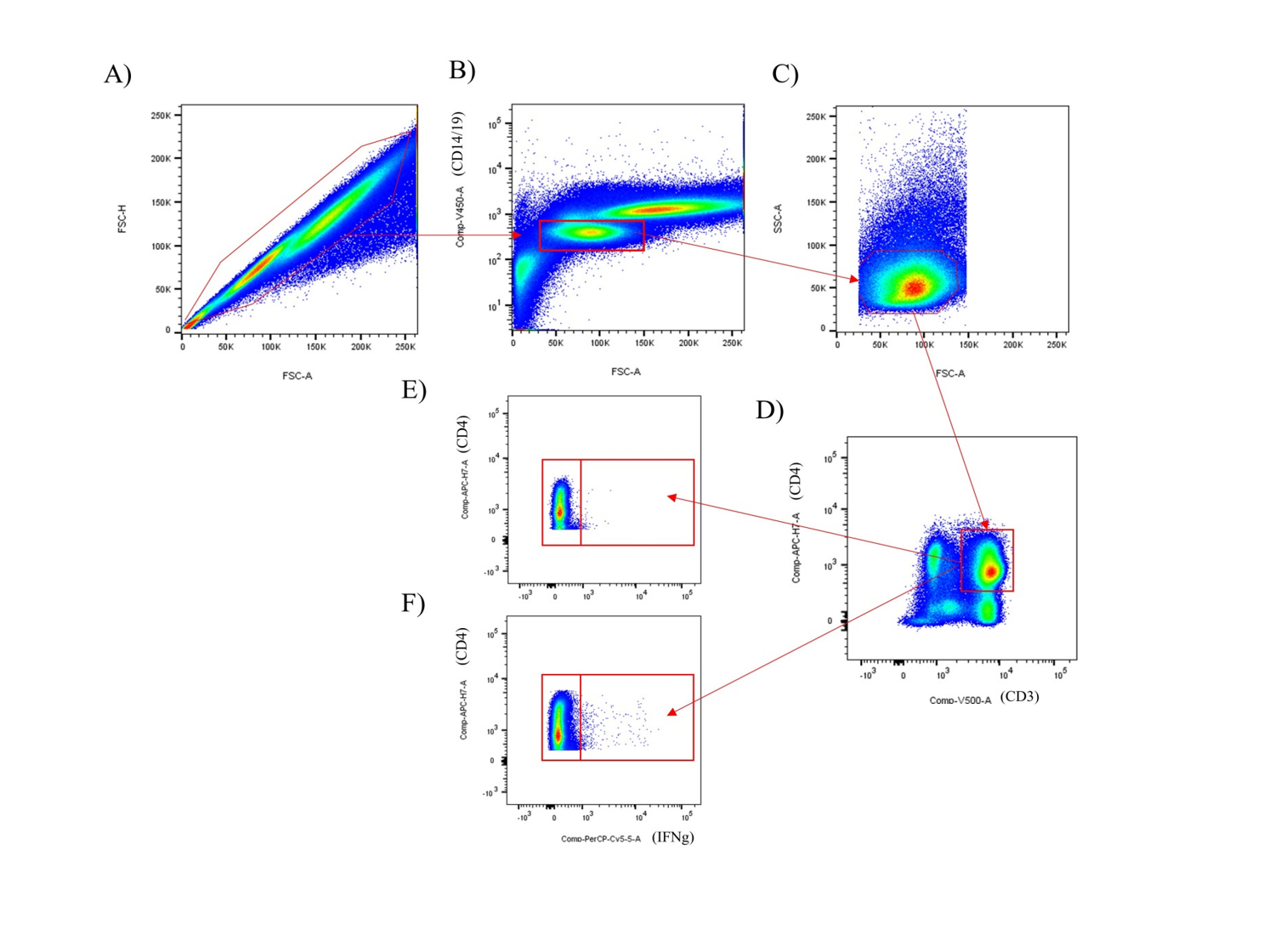
**

**Supplementary Figure 2.** The strategy for gating polyfunctional CD4+ T cells. Gating of singlet **(A)**, negative (i.e. CD14-, CD19-) **(B)**, lymphoid **(C)**, and CD3+CD4+ **(D)** T cells was performed in sequence for each sample. Cytokine gates were then set on unstimulated tubes **(E)** and copied to stimulated tubes **(F)**.

**
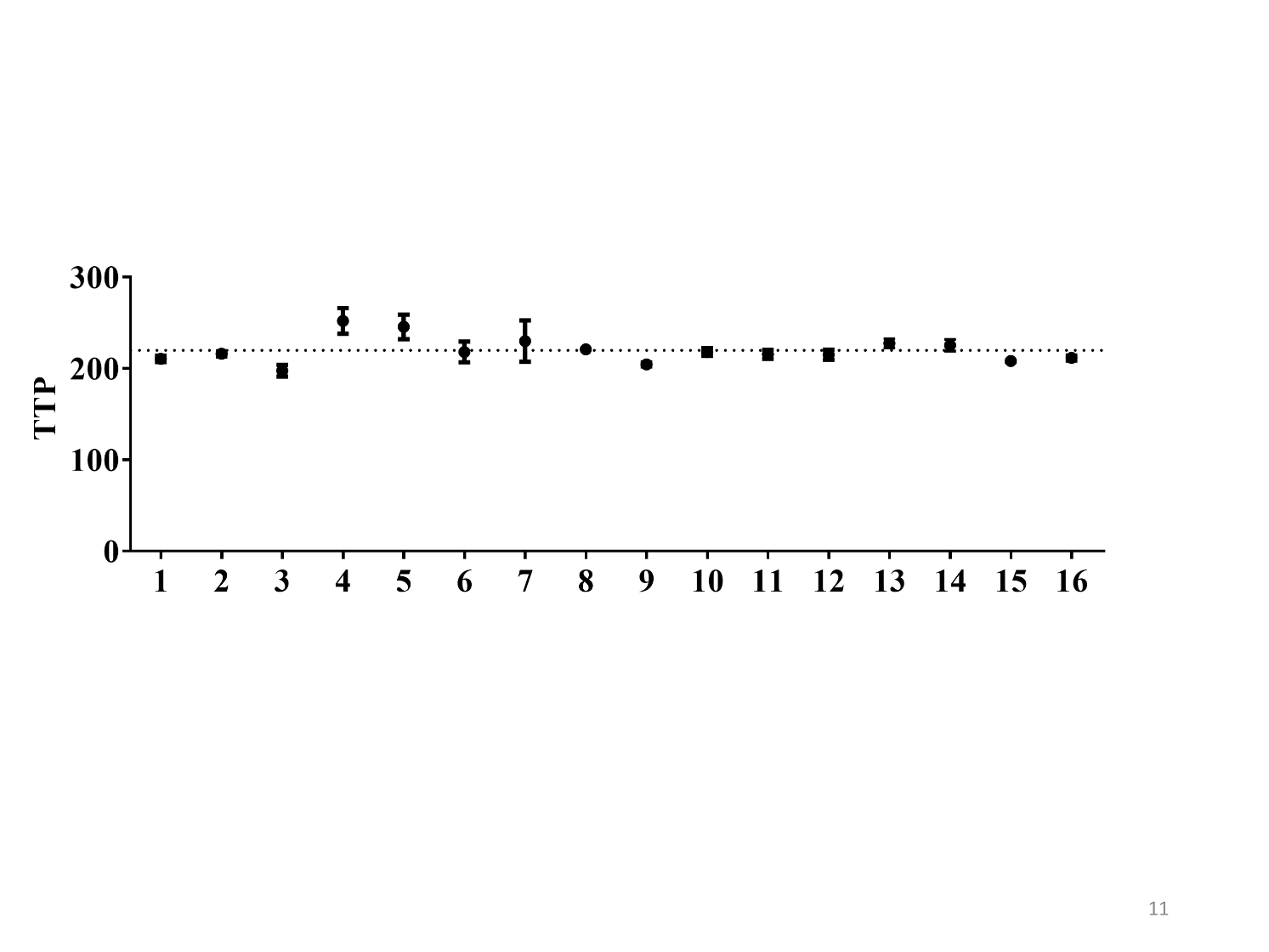
**

**Supplementary Figure 3.** Variation in TTP (in hours) of direct to MGIT control tubes. For MGIA assays, mycobacterial growth in each tube was determined by time to positivity (TTP) in hours. MGIA assays were carried out 16 times and direct-to-MGIT controls were used each time. Mean TTP values of direct to MGIT tubes for the 16 experiments was 219.7 hours. To examine the repeatability and intermediate precision, each TTP of direct-to MGIA controls per experiment were plotted for the 16 experiments. The intra-assay (within-run precision or repeatability) and inter-assay (between-run precision or inter precision) precision coefficients of variation (% CV) were determined to be 2.92 % and 6.44 %, respectively.

**
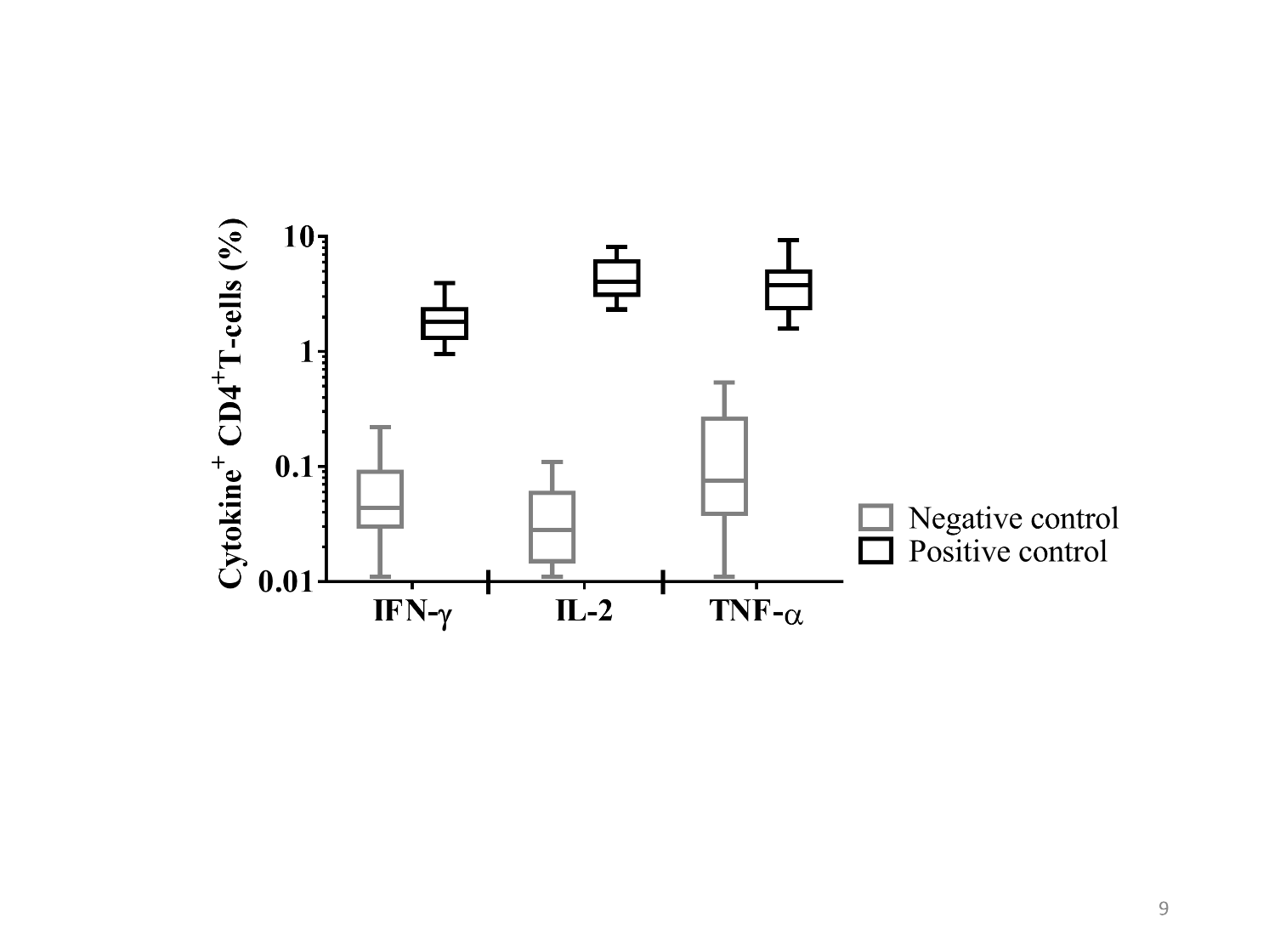
**

**Supplementary Figure 4.** Intracellular cytokine staining assay for negative (light grey) and positive (SEB-stimulated; dark grey) control stimulations of all blood samples.

**
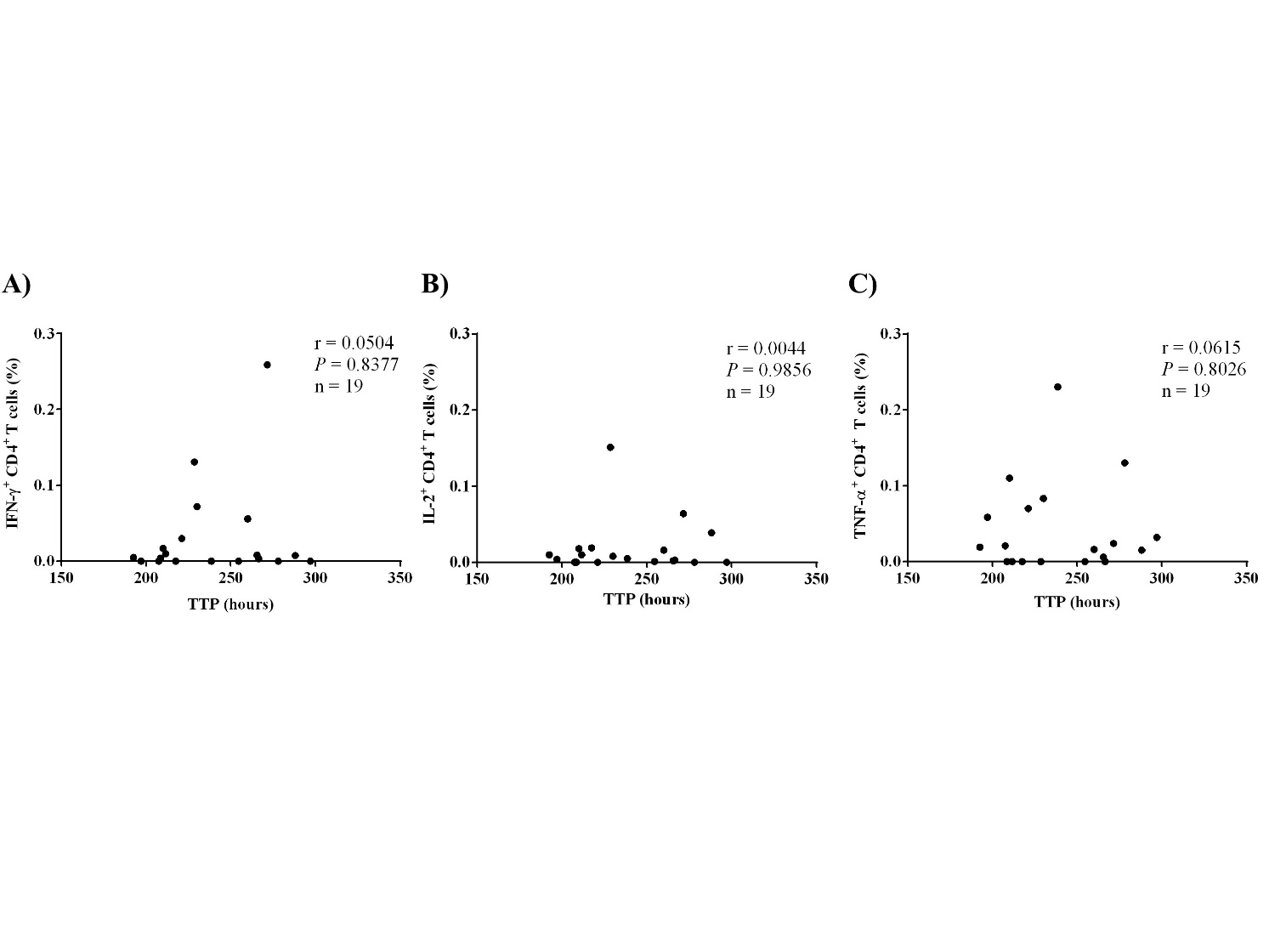
**

**Supplementary Figure 5.** Correlation between mycobacterial growth inhibition and CD4+ T-cells producing single cytokines (IFN-γ, IL-2, and TNF-α). Scatter plots of TTP in hours versus the percentage of CD4+ T-cells producing single cytokines (IFN-γ (A), IL-2 (B), and TNF-α (C)) were drawn for all samples where both the mycobacterial growth inhibition assay and the ICS assay were performed (n = 19). Spearman’s rank correlation coefficient was calculated between growth inhibition and ICS data, indicated as r.
